# Transcriptome Comparison Reveals the Difference in Liver Fat Metabolism between Different Sheep Breeds

**DOI:** 10.3390/ani12131650

**Published:** 2022-06-27

**Authors:** Taotao Li, Meilin Jin, Xiaojuan Fei, Zehu Yuan, Yuqin Wang, Kai Quan, Tingpu Wang, Junxiang Yang, Maochang He, Caihong Wei

**Affiliations:** 1 Key Laboratory of Animal Genetics and Breeding and Reproduction, Ministry of Agriculture, Institute of Animal Sciences, Chinese Academy of Agricultural Sciences, Beijing 100193, China; ltt_ltt2020@163.com (T.L.); jmlingg@163.com (M.J.); 18409481571@163.com (X.F.); 2 Joint International Research Laboratory of Agriculture and Agri-Product Safety, Ministry of Education, Yangzhou University, Yangzhou 225009, China; yuanzehu@yzu.edu.cn; 3 College of Animal Science and Technology, Henan University of Science and Technology, Luoyang 471023, China; wangyq6836@163.com; 4 College of Animal Science and Technology, Henan University of Animal Husbandry and Economy, Zhengzhou 450046, China; quankai1115@163.com; 5 College of Bioengineering and Biotechnology, Tianshui Normal University, Tianshui 741000, China; wangtp002@163.com; 6 Gansu Institute of Animal Husbandry and Veterinary Medicine, Pingliang 744000, China; gsyangjunxiang@163.com (J.Y.); myshmc@163.com (M.H.)

**Keywords:** sheep, liver, fat metabolism, RNA-seq

## Abstract

**Simple Summary:**

Fat metabolism is an important research topic in sheep, and tail fat tissue is usually used as the study material in the fat metabolism of sheep. The liver is also a major fat metabolism organ, and there are many studies on fat metabolism in the livers of chickens, ducks, pigs, and cattle, but studies on the livers of sheep are still fairly rudimentary. In this study, we investigated the liver transcriptome difference between Hu sheep and Tibetan sheep. We reported the differentially expressed genes in the livers of two sheep breeds using RNA-seq technology and identified fat-metabolism-related genes, including *ACSLs*, *CPT1s*, and *FGF21*. We further verified the relative expression levels of genes that are significant and stably expressed on liver fat metabolism at the mRNA and protein levels; then, we described the details of how these genes regulate fat metabolism. The results revealed the difference in fat metabolism between the two sheep breeds.

**Abstract:**

Hu sheep and Tibetan sheep are two commonly raised local sheep breeds in China, and they have different morphological characteristics, such as tail type and adaptability to extreme environments. A fat tail in sheep is the main adipose depot in sheep, whereas the liver is an important organ for fat metabolism, with the uptake, esterification, oxidation, and secretion of fatty acids (FAs). Meanwhile, adaptations to high-altitude and arid environments also affect liver metabolism. Therefore, in this study, RNA-sequencing (RNA-seq) technology was used to characterize the difference in liver fat metabolism between Hu sheep and Tibetan sheep. We identified 1179 differentially expressed genes (DEGs) (*Q*-value < 0.05) between the two sheep breeds, including 25 fat-metabolism-related genes. Through Kyoto Encyclopedia of Genes and Genomes (KEGG) enrichment analysis, 16 pathways were significantly enriched (*Q*-value < 0.05), such as the proteasome, glutamatergic synapse, and oxidative phosphorylation pathways. In particular, one of these pathways was enriched to be associated with fat metabolism, namely the thermogenesis pathway, to which fat-metabolism-related genes such as *ACSL1*, *ACSL4*, *ACSL5*, *CPT1A*, *CPT1C*, *SLC25A20*, and *FGF21* were enriched. Then, the expression levels of *ACSL1*, *CPT1A*, and *FGF21* were verified in mRNA and protein levels via qRT-PCR and Western blot analysis between the two sheep breeds. The results showed that the mRNA and protein expression levels of these three genes were higher in the livers of Tibetan sheep than those of Hu sheep. The above genes are mainly related to FAs oxidation, involved in regulating the oxidation of liver FAs. So, this study suggested that Tibetan sheep liver has a greater FAs oxidation level than Hu sheep liver. In addition, the significant enrichment of fat-metabolism-related genes in the thermogenesis pathway appears to be related to plateau-adaptive thermogenesis in Tibetan sheep, which may indicate that liver- and fat-metabolism-related genes have an impact on adaptive thermogenesis.

## 1. Introduction

Sheep (*Ovis aries*) is one of the important livestock species which provides meat, milk, and wool for human consumption. Under the effects of long-term natural selection, sheep have evolved into different ecotypes, which can be divided into fat-tailed and thin-tailed sheep according to tail type [1]. Hu sheep and Tibetan sheep are two commonly raised local sheep breeds in China, with different tail types and environmental adaptability. The fat tail of sheep is mainly used to store fat, which can provide energy during migration, drought, and food deprivation [2]. Fat is the main energy substance, and as a major energy source, not only should we pay attention to its deposition in fat tissue, but its metabolization in the liver is equally significant. The liver is a central hub for fat metabolism, with the uptake, esterification, oxidation, and secretion of fatty acids (FAs) all occurring in hepatocytes [3]. FAs in the liver can be converted to triglyceride (TG) and cholesterol ester to be secreted as very low-density lipoprotein (VLDL) particles, or they can be eliminated by oxidation [4,5]. A fat tail as a body reserve plays an important role in fat storage in sheep. The amount of fat deposition in adipose tissue is influenced by the amount of fat flux in the liver used for biosynthesis. Characterization of the fat metabolism in sheep liver may reflect the regulatory mechanism behind the differences in phenotype, including the difference in fat tails and thin tails. 

RNA sequencing (RNA-seq) is a revolutionary new tool in the post-genomic era. This contributes not only to the quantification of gene expression, but also to a better understanding of the biological function and role of genes [6]. Recent studies have used RNA-seq to identify the transcriptomes of mouse [7], chicken [8], cow [9], and pig [10,11] livers, which identified fat-metabolism-related genes. However, complete RNA-seq studies of sheep liver metabolism are still fairly rudimentary, especially when compared between different breeds. Ren et al. found that overgrazing caused differences in gene abundance in the livers of sheep through transcriptome analysis. Low feed efficiency and quality result in altered energy metabolism (i.e., lipid metabolism) and changes in detoxification and immune response, resulting in impaired lipid breakdown and health status, ultimately reducing sheep growth. Zhang et al. [12] characterized the liver transcriptome of sheep and analyzed the differentially expressed genes (DEGs) of Mongolian sheep and Lanzhou big-tail sheep. Although both breeds are Mongolian sheep from cold and dry areas in western China, there are differences in their liver transcriptomes.

Considering the unique breed characteristics of Hu sheep and Tibetan sheep from diverse genetic backgrounds, such as the tail phenotype (fat tail vs. thin tail) and the adaptability to the cold environment (Hu sheep live at an altitude of 1500 m, while Tibetan sheep live in alpine areas with altitudes of 5000 m), comparing their livers’ transcription features may help us explore the differences in fat metabolism. So, in this study, RNA-seq was utilized to identify DEGs and pathways regulating fat metabolism in the livers of Hu sheep and Tibetan sheep. We preliminarily characterized the DEGs regarding FA degradation in sheep liver and found that they were significantly enriched in the thermogenesis pathway. The result may indicate the difference in liver fat metabolism between Hu sheep and Tibetan sheep, and it may affect thermogenesis.

## 2. Materials and Methods

### 2.1. Ethical Statement

All experiments involving animals were authorized by the Animal Ethics Committee of the Institute of Animal Science, Chinese Academy of Agricultural Sciences (IAS-CAAS) (Beijing, China). Ethical approval on animal survival was given by the animal ethics committee of IAS-CAAS (No. IAS 2020-82).

### 2.2. Animals and Sample Collection

Six healthy newborn male lambs with similar body weights (3.48 ± 0.78 kg) were used in this study, including three Hu sheep from Yongdeng, Gansu province, and three Tibetan sheep from Yushu, Qinghai province. They were fed breast milk freely until they were weaned at 2 months of age, and then, they were raised under the same environmental conditions with the same nutrition and free access to food and water. After being fed for 16 months, the live weight of Hu sheep and Tibetan sheep was 52.03 ± 1.60 kg and 48.72 ± 1.12 kg, respectively. After the animals were slaughtered, the carcass weight of Hu sheep and Tibetan sheep was 24.98 ± 0.67 and 21.85 ± 0.25, respectively. Then, the liver was sampled rapidly from the left lobe of each animal’s liver and immediately frozen in liquid nitrogen. All tissue samples were stored at −80 °C until they were used for total RNA extraction. 

### 2.3. RNA Extraction, Library Preparation, and Sequencing

Total RNA was extracted using TRIzol (Invitrogen, Carlsbad, CA, USA) following the manufacturer’s instructions. A NanoDrop2000 spectrophotometer was used to quantify RNA purity at 260 and 280 nm (Thermo Fisher Scientific, Waltham, MA, USA). The integrity of RNA and the library was examined using an Agilent 2100 Bioanalyzer (Agilent Technologies, Santa Clara, CA, USA). For sequencing, mRNA with Poly (A) of livers (six samples) was enriched from total RNA using oligo (dT) magnetic beads [13,14]. First-strand cDNA was synthesized using random N6 primers, followed by second-strand cDNA synthesis. The ligation products were amplified using PCR to build a cDNA library, the purified PCR product was denatured into a single strand, and then, the single strand DNA was cycled with primer to generate a single-strand circular DNA library. All libraries were sequenced on the BGISEQ-500 platform (BGI, Shenzhen, China). Six libraries were named HG1, HG2, HG3, ZG1, ZG2, and ZG3. HG represents Hu Sheep liver and ZG represents Tibetan sheep liver. The clean reads were submitted to the Sequence Read Archive (accession numbers PRJNA792689).

### 2.4. Quality Control and Mapping of Reads

The raw reads with low-quality reads, adaptors, and highly unknown base N content were trimmed using SOAPnuke, a filtering software developed by BGI. Then, clean reads were aligned to the sheep reference genome of *Ovis aries* (Oar_v3.1) using HISAT2 (v2.1.0). We selected HISAT2 as the mapping tool because it has a fast operation speed, high sensitivity, and low memory consumption.

### 2.5. Identification of DEGs

Fragments per kilobase million (FPKM) values were used to characterize the expression levels of genes, which consider the effect of sequencing depth and gene length on reading counts at the same time and allow for the direct comparison of gene expression differences between two samples [15]. The expression levels of genes were estimated using RSEM v1.2.8 [16], which is a software package for RNA-seq reads used to calculate gene and transcription isoforms. 

To compare the expression levels of genes across samples, differential expression analyses between different samples were performed using DESeq2 [17,18]. Genes with |log2Fold Change| > 0.5 and *Q*-values < 0.05 were recognized as DEGs.

### 2.6. Functional Annotation, and Pathway Enrichment Analysis of DEGs

To further understand the potential functions of the DEGs, all DEGs were mapped to Gene Ontology (GO) terms and Kyoto Encyclopedia of Genes and Genomes (KEGG) pathways, which were performed using the phyper package in R. After FDR correction, a *Q*-value < 0.05 was used as a threshold. All analyses with a *Q*-value < 0.05 were considered significantly enriched.

### 2.7. Quantitative Reverse–Transcription PCR (qRT-PCR) for DEGs Verification

Ten genes were randomly selected for qRT-PCR to verify the accuracy of the transcriptome sequencing data. The total RNA of the liver tissue was extracted using TRIzol reagent (Invitrogen, Carlsbad, CA, USA), and then, about 0.1 µg of RNA was reverse transcribed into cDNA using a PrimerScript RT reagent Kit (TaKaRa, Tokyo, Japan). The synthesized cDNA was used as the template for qRT-PCR using SYBR Premix Ex Taq II by the LightCycler 480II (Roche, Basel, Sweden). The procedure involved 40 cycles of pre-denaturation at 95 °C for 5 s; denaturation at 95 °C for 5 s and at 60 °C for 30 s. There were triplicates in all experiments, and β-actin was used as an internal reference gene. The primer sequences are listed in Table S1. After the reaction was completed, a melting curve analysis was performed. The 2^−ΔΔCT^ method was used to analyze the changes in relative gene expression. 

### 2.8. Western Blotting for Fat Metabolism Related Genes

Protein lysates were prepared from the liver tissue using the RIPA lysis buffer (Beyotime, Shanghai, China), and the protein concentration was measured using the BCA protein detection kit. Then, 20 μg protein samples were denatured at 95 °C for 5 min. After SDS-PAGE electrophoresis, the proteins were transferred onto a PVDF membrane at 200 mA for 90 min. The membrane was blocked at room temperature with a quick blocking buffer (SunBioo, Beijing, China) for 10 min. Subsequently, the membranes were incubated with Anti-ACSL1 (1:3000, 13989-1-AP, Proteintech, Chicago, IL, USA), Anti-CPT1A (1:2000, 15184-1-AP, Proteintech, Chicago, IL, USA), Anti-FGF21 (1:1000, ab171941, Abcam, Cambridge, UK), and Anti-GAPDH (1:1000, ab56416, Abcam, Cambridge, UK) at 4 °C overnight. After washing, horseradish peroxidase-conjugated anti-rabbit antibodies were added for further incubation, and then, the signal was developed using a chemiluminescent imaging system and quantified using ImageJ software. Photoshop CC 2019 was used to crop images from unprocessed images.

## 3. Results

### 3.1. Sequencing and Mapping of Reads

A total of 266.42 million raw reads for six samples were obtained through sequencing. After quality control, an average of 43.62 million clean reads were generated (43.14 million to 43.22 million for Hu sheep and 43.05 million to 44.65 million for Tibetan sheep). The percent of clean reads was ≥97.7% for each sample. The Q30 values were between 91.83% and 93.55%, which met the requirement that the sequence Q30 had to be over 90%. 

The clean reads were mapped to the sheep reference genome. An approximate average of 86% of the clean reads were mapped to the *Ovis aries* reference genome (Hu sheep with 86.25%, Tibetan sheep with 85.92%), and 42.83% to 50.58% of the clean reads were uniquely mapped to the *Ovis* *aries* reference genome. These data were suitable for further analysis of the DEGs. Table 1 shows the basic statistics for RNA-seq reads generated from the livers of Hu sheep and Tibetan sheep.

### 3.2. Analysis of Gene Expression

**Figure 1:**
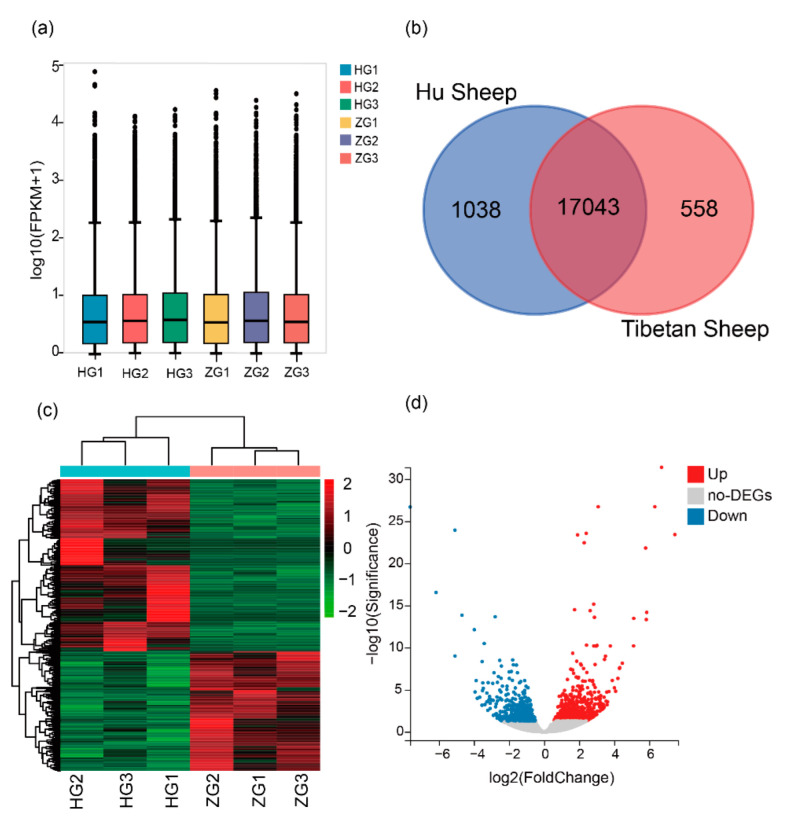
(**a**) Box plot of FPKM distribution in six samples. The abscissa represents the sample name, and the ordinate represents log10(FPKM+1). HG1, HG2, and HG3 are representative of the Hu sheep liver. ZG1, ZG2, and ZG3 are representative of Tibetan sheep liver. (**b**) Venn diagram of genes expressed in the livers of Hu sheep and Tibetan sheep. (**c**) Heat map of the DEGs cluster analysis. The different columns represent different samples, with different rows representing different genes. (**d**) Volcano plot displaying DEGs (*Q*-value < 0.05) in the livers of Hu sheep and Tibetan sheep. Up, up-regulated DEGs; Down, down-regulated DEGs.

To better understand the difference in liver metabolism between Hu sheep and Tibetan sheep, DEGs in the two breeds were identified. According to the box plot of FPKM distribution, the gene expression distributions in Hu sheep were similar to Tibetan sheep (Figure 1a). According to the FPKM values, the largest proportions of genes were expressed at medium to high (≥10 FPKM) and lower (from 1 FPKM to 10 FPKM) expression levels, and only a small fraction were expressed at very low expression levels (≤1 FPKM). In total, we identified 18,638 expressed genes (FPKM > 0) in two sheep breeds. Of these, 1038 genes were only expressed in Hu sheep, 558 genes were only expressed in Tibetan sheep, and 17,043 genes were expressed in both sheep breeds (Figure 1b). Then, a total of 1179 DEGs (*Q*-value < 0.05) were identified between two sheep breeds, with 693 genes up-regulated and 486 genes down-regulated in the livers of Hu sheep (Figure 1c,d; Table S2).

### 3.3. Enrichment Analysis of DEGs

**Figure 2:**
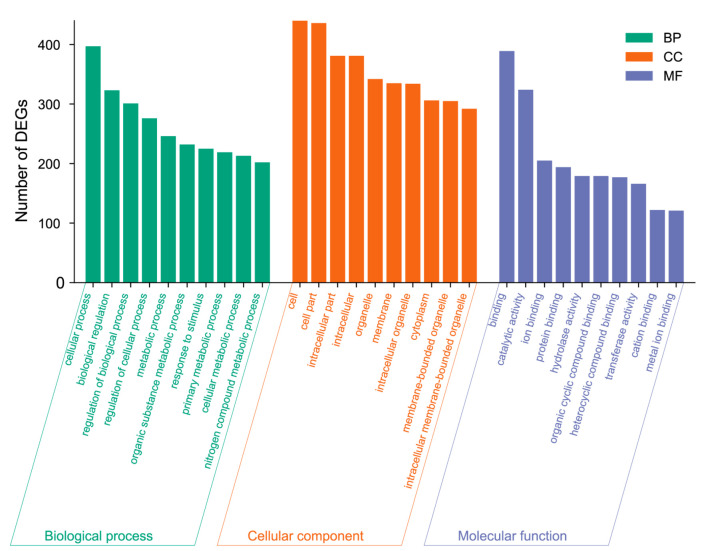
GO enrichment analyses of the DEGs in livers of Hu sheep and Tibetan sheep. The GO terms include biological processes (BPs), cellular components (CCs), and molecular functions (MFs).

In total, 785 DEGs were enriched to GO terms. GO annotation has three classifications: biological processes (BPs), cellular components (CCs), and molecular functions (MFs), which classify DEGs (Figure 2). In total, 30 terms were significantly enriched in BPs (*Q*-value < 0.05), including “small molecule metabolic process”, “regulation of response to external stimulus”, and “energy derivation by oxidation of organic compounds”. Overall, seven terms were significantly enriched in CCs (*Q*-value < 0.05), such as “mitochondrion”, “proteasome regulatory particle”, and “proteasome accessory complex”, and three terms were significantly enriched in MFs (*Q*-value < 0.05), including “endopeptidase regulator activity”, “proteasome-activating ATPase activity”, and “cysteine-type endopeptidase activity involved in the apoptotic process” (Table S3). No fat-metabolism-related terms were significantly enriched; however, we found several fat-metabolism-related genes significantly enriched in the catabolic process, the organic substance catabolic process, and the small molecule metabolic process.

**Figure 3:**
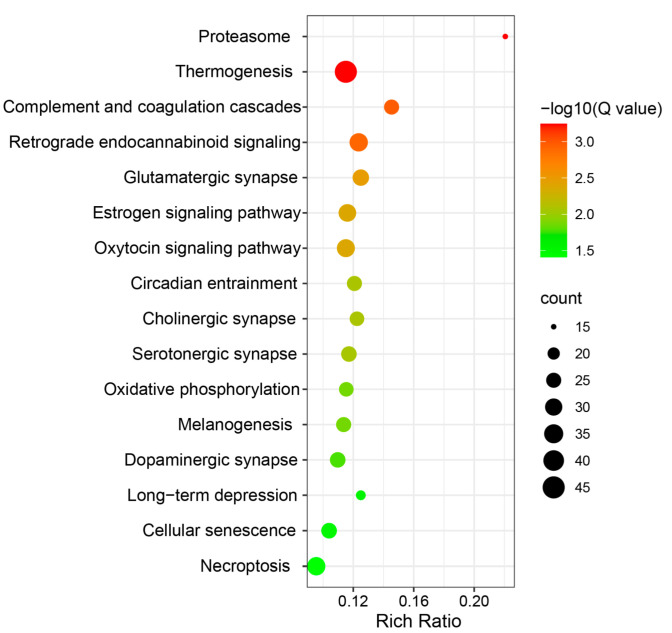
Significantly enriched KEGG pathways of DEGs (*Q*-value < 0.05). The abscissa represents the rich ratio, and the ordinate represents the pathway name.

**Figure 4:**
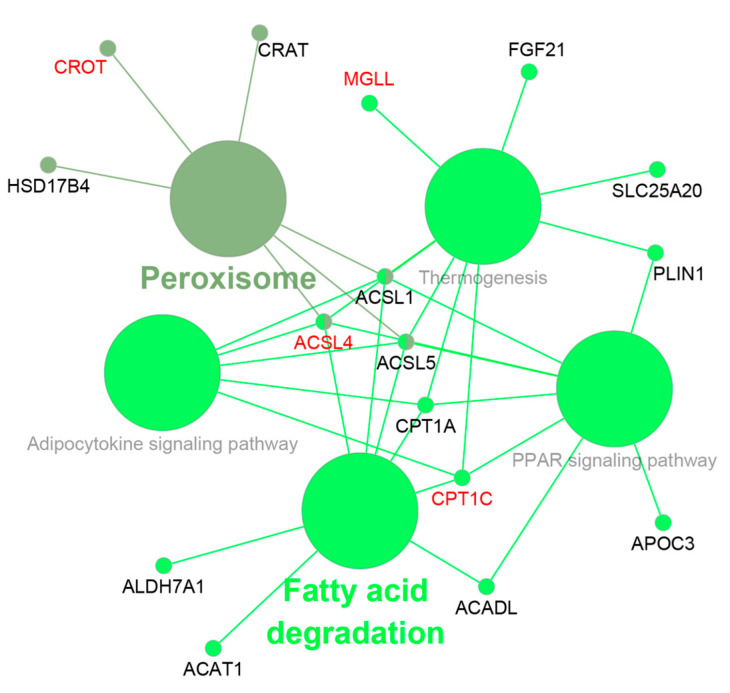
ClueGo analysis of fat-metabolism-related genes. The red represents the up-regulated genes, and the black represents the down-regulated genes.

Next, the DEGs were analyzed using the KEGG database to identify enriched pathways. In total, 718 DEGs had KEGG pathway annotations. A total of 16 pathways were significantly enriched (*Q*-value < 0.05) (Figure 3), including proteasome, thermogenesis, complement and coagulation c, glutamatergic synapse, cholinergic synapse, oxidative phosphorylation, monobactam biosynthesis, and long-term depression. Through GO and KEGG functional enrichment analyses, 25 fat-metabolism-related DEGs were identified in the livers of Hu sheep and Tibetan sheep (Table 2). Among these genes, 9 genes (*ACSL4*, *CPT1C*, *AKR1B1*, *SOAT1*, *PLA2G4A*, *TTC39A*, *PPT1*, *CROT,* and *LOC10110561*) were up-regulated, and 16 genes (*ACSL1*, *ACSL5*, *CPT1A*, *CRAT*, *ACADL*, *SLC25A20*, *ACAT1*, *FGF21*, *APOC3*, *PLIN1*, *ALDH7A1*, *HSD17B4*, *LCAT*, *SOAT2*, *LOC101122246,* and *MGLL*) were down-regulated. Then, we used ClueGo for the enrichment analysis of these 25 genes and found that they were mainly enriched in peroxisome and FA-degradation-related pathways (Figure 4), including thermogenesis and PPAR signaling pathways.

### 3.4. RNA-seq Data Validation by qRT-PCR

**Figure 5:**
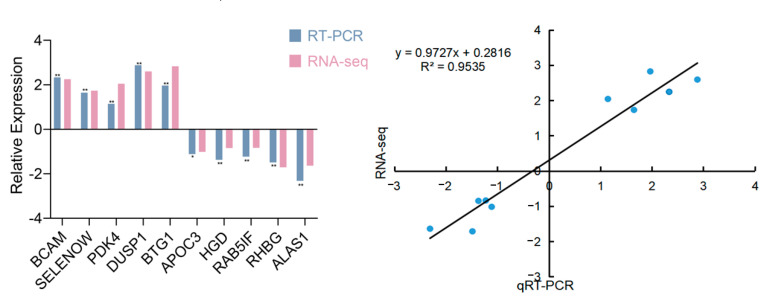
Validation of RNA-Seq results via Quantitative Reverse–Transcription PCR (qRT-PCR). The significant differences (* *p* < 0.05; ** *p* < 0.01) were performed using two-tailed unpaired *t*-test.

To validate the RNA-seq data, 10 DEGs were selected to detect expression levels using qRT-PCR. The expression levels of these genes in the livers of Hu sheep and Tibetan sheep were verified. As shown in Figure 5, *BCAM*, *SELENOW*, *PDK4*, *DUSP*, and *BTG1* were up-regulated in the livers of Hu sheep, and *APOC3*, *HGD*, *RAB5IF*, *RHBG*, and *ALAS1* were down-regulated in the livers of Hu sheep. Pearson correlation analysis suggested that the qRT-PCR results were consistent with the transcriptome sequencing results (R^2^ = 0.9535). 

### 3.5. Analysis of CPT1A, ACSL1, and FGF21 Expression Levels

**Figure 6:**
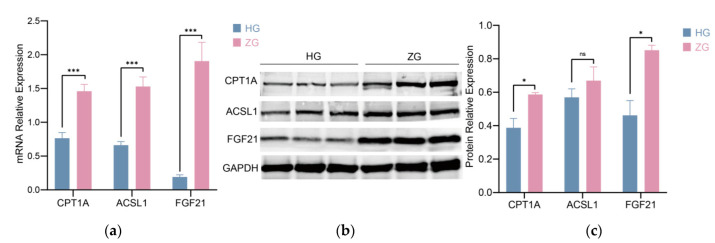
The relative expressions of *CPT1A*, *ACSL1*, and *FGF21* in Hu sheep and Tibetan sheep livers via qRT-PCR (**a**) and Western blot analysis (**b**,**c**). HG is representative of the Hu sheep livers. ZG is representative of Tibetan sheep livers. The data in (**a**,**c**) are presented as the mean ± SD, *n* = 3 biologically independent samples; the significant differences (n.s. *p* > 0.05; * *p* < 0.05; *** *p* < 0.001) were performed using two-tailed unpaired *t*-test.

*ACSL1* and *CPT1A* are the major genes in FA oxidation as they activate and transport long-chain FAs, respectively. *FGF21* regulates fat metabolism and energy homeostasis. The three genes were also significantly enriched in thermogenesis pathways. So, we selected these three genes to further validate their relative expression at the mRNA and protein levels. Compared with Hu sheep, the *CPT1A* and *FGF21* expressions were significantly high at the mRNA (*p* < 0.001) and protein (*p* < 0.05) levels in the livers of Tibetan sheep (Figure 6 and Figures S1–S4; Table S4); the results further illustrated the difference in fat metabolism between Hu sheep and Tibetan sheep.

## 4. Discussion

The mechanisms of fat metabolism are complex. Unlike fat tissue, the liver, as a metabolic center, is primarily involved in fat metabolism, not just fat deposition. In this study, 1179 genes were found to be differentially expressed (693 were up-regulated and 486 were down-regulated) in a DEGs analysis between Hu sheep and Tibetan sheep. The reliability of the transcriptome data was validated using qRT-PCR. Through the GO and KEGG enrichment analyses of DEGs, fat-metabolism-related genes were screened from GO terms and KEGG pathways, including *ACSL1*, *ACSL4*, *ACSL5*, *CPT1A*, *CPT1C*, *PLIN1*, *HSD17B4*, *ACAT1*, *ACADL*, *FGF21*, and so on. We also further validated the reliability of three fat-metabolism-related genes via qRT-PCR and Western blot. The above genes are mainly involved in the regulation of FA degradation, including the activation and oxidation processes. 

### 4.1. Fatty Acid Activation

**Figure 7:**
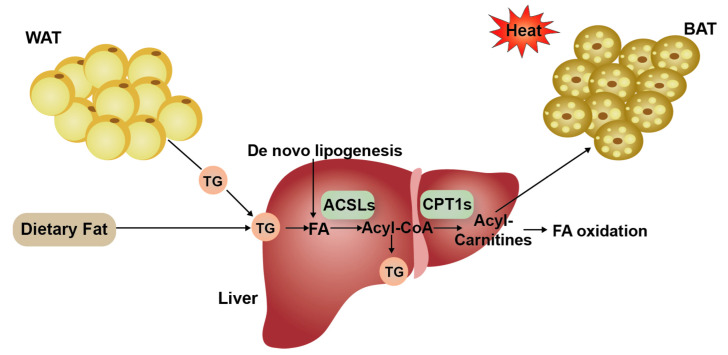
Schematic diagram of liver fatty acid (FA) oxidation. Liver FAs are mainly derived from dietary lipids, adipose tissue decomposition, and de novo synthesis. FAs are activated by long-chain acyl-CoA synthetases (ASCL) to form acyl-CoA molecules, which can undergo oxidation or be incorporated into complex lipids. Acyl-CoA is transferred to mitochondria by carnitine palmitoyltransferase 1 (CPT1), and then, Acyl-CoA converts Acyl-Carnitines, which can partition to brown adipose tissue (BAT) for heat generation or continually into FA oxidation.

The activation of long-chain FAs is the first step in their usage in cells and tissues [19]. FAs are first activated into acyl-CoA and then integrated into metabolic pathways such as β-oxidation or complex lipid biosynthesis. This reaction is catalyzed by members of the long-chain acyl-CoA synthetase (ACSL) family of enzymes (Figure 7) [5,20]. There are five *ACSL* isoforms present in mammals, namely *ACSL1*, *ACSL3*, *ACSL4*, *ACSL5*, and *ACSL6*, with different distributions, substrate preferences, and specific functions [21]. Additionally, distinct *ACSL* isoforms play key roles in partitioning long-chain fatty acyl-CoAs into different metabolic pathways [22,23]. *ACSL1* is highly expressed in the liver, muscle, and adipose tissue [24] and is mainly located in mitochondria and the endoplasmic reticulum. Previous studies have found that *ACSL1* in the liver is a target of peroxisome proliferator activated receptor alpha (PPARα), which enhances the expression of genes involved in β-oxidation [25]. However, some data have indicated that deficiencies in *ACSL1* reduced the synthesis of TG and FAs β-oxidation [23]. It has also been reported that the over-expression of *ACSL1* leads to the production and accumulation of large amounts of TG in hepatoma cells [26]. In our study, greater expression of *ACSL1* in the livers of Tibetan sheep was found to consist of the expression in the tail fat tissue of the Zel (thin-tailed) sheep breed vs. the Lori-Bakhtiari (fat-tailed) sheep breed [27], which seems to indicate that *ACSL1* preferentially partitions to oxidative rather than deposition pathways. *ACSL4* is enriched in the adrenal gland and liver, and *ACSL5* is widely expressed in brown adipose tissue (BAT), the small intestine, and the liver [28]. *ACSL4* prefers polyunsaturated fatty acid (PUFA) as its substrates [19], such as arachidonic acid and eicosapentaenoic acid. Transcriptome analysis of the mammary glands of Holstein cows found that the up-regulation of *ACSL4* increased the TG concentration, while its down-regulation decreased the TG concentration [29]. *ACSL5* is the only *ACSL* isoform located in mitochondria and participates in lipid anabolism, which can activate and guide FAs into anabolism pathways, such as promoting lipid droplet formation in TG synthesis and storage [30,31]. Thus, the differential expression of *ACSL1*, *ACSL4*, and *ACSL5* between Hu sheep and Tibetan sheep indicated the difference in FA activation in the two sheep breeds’ livers. Specific to the role of each *ACSL* isoform, the up-regulation of *ACSL4* and the down-regulation of *ACSL1* and *ACSL5* seemed to suggest that FA activation in the livers of Tibetan sheep is more integrated into the oxidation process than that of Hu sheep.

### 4.2. Fatty Acid Oxidation

After activation in the cytoplasm, FAs are transferred to mitochondria and peroxisome for oxidation. Carnitine acetyltransferases catalyze the conversion of fatty acyl-CoAs into acyl-carnitines (Figure 7), the first step in the transport of FAs from the cytoplasm to the mitochondrial matrix and peroxisome, where they undergo β-oxidation [32,33]. The above steps are essential, as the mitochondrial inner membrane is not permeable to long-chain fatty acid acyl-CoAs. Carnitine acyltransferases include CRAT, CROT, CPT1, and CPT2, and they have a wide range of acyl chain specificity [34]. CRAT displays a preference for short-chain acyl-CoA esters (such as acetyl-CoA). CROT is a peroxisomal enzyme that preferentially catalyzes medium-chain acyl-CoAs [35], including branched FAs such as phytanic acid, present in the diet. Medium and long acyl-CoA chains require CPT1 and CPT2 to enter the mitochondrial matrix and proceed to the β-oxidation cycles. Compared to other members, CPT1, as the key rate-limiting enzyme in the mitochondrial oxidation of long-chain FAs, plays an important role in catalyzing the conversion of long-chain fatty acyl-CoAs into acyl-carnitines [33]. The three CPT1 isoforms are CPT1A, CPT1B, and CPT1C, which have different substrate preferences and physiological functions. CPT1A is the liver isoform of CPT1. A lack of CPT1A in the liver results in the failure to generate acyl-carnitines that enter the mitochondria [32], which in turn leads to a low capacity for FA oxidation and secondary gluconeogenesis impairment. CPT1C is the brain isoform of CPT1 and localizes to the endoplasmic reticulum. Studies have found that CPT1C is involved in the biosynthesis process to ensure energy homeostasis and weight control, rather than regulating FA oxidation [36,37,38]. The SLC25A20 transporter, also known as carnitine acyl-carnitine carrier (CACT), is involved in the transport of acyl-carnitines into the mitochondrial matrix for oxidation [39]. Thus, the CROT, CRAT, CPT1A, CPT1C, and SLC25A20 proteins play roles in fat metabolism and FA oxidation. The down-regulation of *CRAT*, *CPT1A*, and *SLC25A20* and the up-regulation of *CROT* and *CPT1C* in Hu sheep vs. Tibetan sheep suggested that Tibetan sheep have more FAs in the oxidation pathway. In addition, the expressions of *ACADL*, *ACAT1*, and *FGF21* were down-regulated in the livers of Hu sheep. *ACADL* encodes a mitochondrial matrix enzyme, LCAD, catalyzing the first step for the oxidation of long-chain fatty acyl-CoAs [40,41,42]. Additionally, *ACAT1* encodes the enzyme catalyzing the last step of long-chain FAs β-oxidation in the mitochondria matrix [43]. FGF21 is a hormone that has been shown to play a key role in the regulation of energy homeostasis, insulin sensitivity, and ketogenesis in both preclinical studies and men [44,45,46,47]. It is produced predominantly in the liver but also in WAT, BAT, the pancreas, and skeletal muscle [48]. In the liver, *FGF21* is induced by PPARα [49,50]. *FGF21* has dramatic effects on liver metabolism that include the induction of FA oxidation, ketogenesis and gluconeogenesis, and the suppression of lipogenesis [51]. Taken together, these data suggest that the liver FA oxidation level of Tibetan sheep was greater when compared with Hu sheep. 

The coupling of FA oxidation and oxidative phosphorylation in mitochondria is the most important pathway for the production of metabolic energy. Mitochondria are at the center of energy metabolism, and they coordinate energy metabolism through five oxidative phosphorylation (OXPHOS) protein complexes [52]. NADH and FADH2 produced during FA oxidation pass their electrons to the OXPHOS complexes to generate ATP. Therefore, FA oxidation and OXPHOS share substrates and are linked biochemically [53]. The expression of each of the five mitochondrial OXPHOS complexes was lower in subjects with acquired obesity and insulin resistance [52,54]. We discovered the expression of mtDNA, its transcripts, OXPHOS subunits (*ND4*, *ND5*, *ND6*, *ATP6*, *COX3*, *CYTB*, *LOC101121285*, and *LOC101113599*), as well as nuclear genes encoding OXPHOS proteins (*NDUFB7*, *NDUFB9*, *NDUFB10*, *NDUFS3*, *NDUFS7*, *NDUFS8*, *NDUFV1*, *ATP6V0A1*, and *ATP5MC1*) were expressed at a significantly lower rate in the liver of Hu sheep when compared with Tibetan sheep, suggesting a significantly lower expression rate in mitochondrial oxidative ATP production and catabolic functions in Hu sheep than those of Tibetan sheep. This was consistent with the previous statement that FA oxidation in Tibetan sheep was greater than that in Hu sheep. These results suggested that there are different energy metabolisms in the two sheep breeds. 

### 4.3. Thermogenesis Pathway

FA-metabolism-related genes (*ACSL1*, *ACSL4*, *ACSL5*, *CPT1A*, *CPT1C*, *SLC25A20*, and *FGF21*) and OXPHOS-protein-complex-encoding genes (*ATP6*, *COX3*, *COX14*, *ND4*, *ND5*, *ND6*, *CYTB*, *ATP6V0A1*, *NDUFB7*, *NDUFB9*, *NDUFB10*, *ATP5MC1*, *NDUFS3*, *NDUFS7*, *NDUFV1*, *LOC101121285*, and *LOC101113599*) were significantly enriched in the thermogenesis pathway. BAT [55] is the main thermogenesis tissue, and it dissipated the energy generated via oxidative phosphorylation as heat [56]. Although the specific function of the liver on thermogenesis is unclear, Simcox et al. [57] found that the liver undergoes a metabolic switch to provide fuel for BAT thermogenesis by producing acyl-carnitines. Cold exposure triggers systemic changes in fat metabolism, which increases FA delivery to BAT by various routes. Brown adipocytes utilize FAs released by WAT for adaptive thermogenesis. WAT-derived FAs are internalized directly by BAT or indirectly after hepatic conversion to VLDL and acyl-carnitines [58]. So, the genes related to acyl-carnitine metabolism and even FA metabolism in the liver could affect thermogenesis, such as *ACSLs* (FA activation), and *CPT1s* (FA oxidation) (Figure 7). *FGF21* was also a top DEG and was enriched in the thermogenesis pathway. An important role of *FGF21* is to regulate interscapular BAT to increase thermogenesis [59]. This role was first proposed in a study using newborn mice in which hepatic *FGF21* expression was increased through maternal milk [60], and then, it increased the thermogenic gene expression of BAT in neonates, and cold exposure also rapidly induced the expression of *FGF21* in the BAT of adult mice, suggesting that *FGF21* plays a role in improving thermogenesis [61,62]. Hence, FGF21 has been identified as a thermogenic hormone to control thermogenesis [63,64]. *FGF21* is associated with energy expenditure and thermogenesis and provides a mechanism for weight loss [65,66]. Therefore, the differential expression of *FGF21* in different sheep breeds may affect fat deposition in adipose tissues and adaptability to cold environments through energy expenditure and thermogenesis. The liver may play an important role in the energy supply of thermogenesis. 

Combined with the above results, this study showed that the mRNA expression pattern of the liver is somewhat different from that of adipose tissue, with the main function of FA oxidation rather than lipogenesis. The lower expression of FA-oxidation-related genes (e.g., *ACSL1*, *ACSL5*, *CPT1A*, *CRAT*, *SLC25A20*, *ACADL*, *ACAT1*, and *FGF21*) in Hu sheep promoted a shift from FA oxidation to lipogenesis in the liver. It seems that there are differences in fat metabolism between adipose tissue and the liver, and such tissue-specific changes might reflect whole-body changes in fat turnover. The Tibetan sheep is a plateau breed, living at an altitude of 5000 m. They need energy generated by FA oxidation to resist the cold environment and maintain their body temperature, which may be biologically relevant to the plateau adaptations of Tibetan sheep, while Hu sheep need a strong fat synthesis ability to deposit tail fat. Therefore, the FA oxidation level in Tibetan sheep is greater than that in Hu sheep. However, in this study, there were only three sheep sample sizes per breed, and the metabolic data were missing, which may be limitations to this study.

## 5. Conclusions

In this study, a total of 1179 DEGs were identified in the livers of Hu sheep and Tibetan sheep, including 25 fat-metabolism-related genes, such as *ACSL1*, *ACSL4*, *ACSL5*, *CROT*, *CRAT*, *CPT1A*, *CPT1C*, *ACADL*, and *FGF21*. These genes mainly regulate FA activation and oxidation. Most of these genes were down-regulated in the liver of Hu sheep. The mRNA and protein expression levels of *ACSL1*, *CPT1A*, and *FGF21* in Tibetan sheep livers were significantly higher than those in Hu sheep livers. The results indicated that Tibetan sheep liver has a greater FA oxidation level. In addition, these genes were also enriched in the thermogenesis pathway, which may indicate the important role of these genes in adaptive thermogenesis and their biological relevance to the plateau adaptations of the liver. This study provided reference values for subsequent studies on the fat metabolism of fat-tailed sheep and thin-tailed sheep. For future study, a comprehensive analysis of the tail adipose tissue and the liver in this two sheep breeds, as well as more complete metabolic studies in the liver, may provide a deeper understanding of fat metabolism in sheep.

## Figures and Tables

**Table 1:** The basic statistics for RNA-seq reads were generated from the livers of Hu sheep and Tibetan sheep.

Sample	Raw Reads (M)	Clean Reads (M)	Clean Reads Q20 (%)	Clean Reads Q30 (%)	Clean Reads Ratio (%)	Total Mapping (%)	Uniquely Mapping (%)
HG1	43.82	43.14	97.50	93.19	98.44	86.32	49.96
HG2	43.82	43.15	97.50	93.19	98.48	86.54	50.31
HG3	43.82	43.22	97.64	93.55	98.64	85.88	50.58
ZG1	43.82	43.05	97.71	93.81	98.25	86.96	45.81
ZG2	45.57	44.53	96.89	92.22	97.70	85.66	45.21
ZG3	45.57	44.65	96.72	91.83	97.98	85.14	42.83

**Table 2:** The fat-metabolism-related DEGs in the livers of Hu sheep vs. Tibetan sheep.

Genes	Description	Expression Up Down	*Q*-Value
*FGF21*	Fibroblast growth factor 21	Down	7.43 × 10^−13^
*ACADL*	acyl-CoA dehydrogenase long chain	Down	3.26 × 10^−^^9^
*LOC10110561*	cytochrome P450 4A24-like	Up	3.84 × 10^−^^24^
*ACSL1*	acyl-CoA synthetase long-chain family member 1	Down	0.00387508
*ACSL4*	acyl-CoA synthetase long-chain family member 4	Up	0.012611264
*ACSL5*	acyl-CoA synthetase long-chain family member 5	Down	0.001482037
*CRAT*	carnitine O-acetyltransferase	Down	0.000712661
*CROT*	carnitine O-octanoyl transferase	Up	0.043104451
*CPT1A*	carnitine palmitoyl transferase 1A	Down	0.036011154
*CPT1C*	carnitine palmitoyl transferase 1C	Up	0.018612916
*ACAT1*	acetyl-CoA acetyltransferase 1	Down	0.020168722
*SLC25A20*	solute carrier family 25 member 20	Down	0.046898311
*ALDH7A1*	dehydrogenase 7 family member A1	Down	0.037881737
*HSD17B4*	Hydroxysteroid 17-beta dehydrogenase 4	Down	0.001502217
*AKR1B1*	aldo-keto reductase family 1 member B	Up	0.040502107
*LCAT*	lecithin-cholesterol acyltransferase	Down	0.031312534
*SOAT1*	sterol O-acyltransferase 1	Up	0.017763434
*SOAT2*	sterol O-acyltransferase 2	Down	0.018783871
*PPT1*	palmitoyl-protein thioesterase 1	Up	0.006656028
*TTC39A*	tetratricopeptide repeat domain 39A	Up	0.000475153
*LOC101122246*	2-acylglycerol O-acyltransferase 3	Down	0.019110951
*PLA2G4A*	phospholipase A2 group IVA	Up	0.006462653
*MGLL*	monoglyceride lipase	Down	0.0023883
*PLIN1*	perilipin 1	Down	0.003473361
*APOC3*	apolipoprotein C3	Down	0.002739368

## Data Availability

The data presented in this study are openly available in Sequence Read Archive at https://www.ncbi.nlm.nih.gov/sra (accessed on 25 April 2022), reference number PRJNA792689.

## Supplementary Materials

The following supporting information can be downloaded at: https://www.mdpi.com/article/10.3390/ani12131650/s1, Table S1: The primer information regarding qRT-PCR, Table S2: The details of differentially expressed genes between Hu sheep and Tibetan sheep, Table S3: Significantly enriched GO terms of DEGs, Table S4: Western blot band intensity analysis, Figure S1: Original Western blot figure of gene *ACSL1*, Figure S2: Original Western blot figure of gene *CPT1A*, Figure S3: Original Western blot figure of gene *FGF21*, Figure S4: Original Western blot figure of gene *GAPDH*.
