# Supplementary material for: Transcriptome Comparison Reveals the Difference in Liver Fat Metabolism between Different Sheep Breeds
Source: Animals (Basel). 2022 Jun 27;12(13):1650. doi: 10.3390/ani12131650 (PMC9265030; doi:10.3390/ani12131650)
Supplement: Supplementary file 1 [file animals-12-01650-s001.zip › Figures S1-S4.pdf]

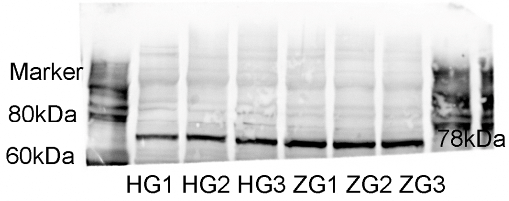

Figure S1. Original Western blot figure of gene ACSL1.

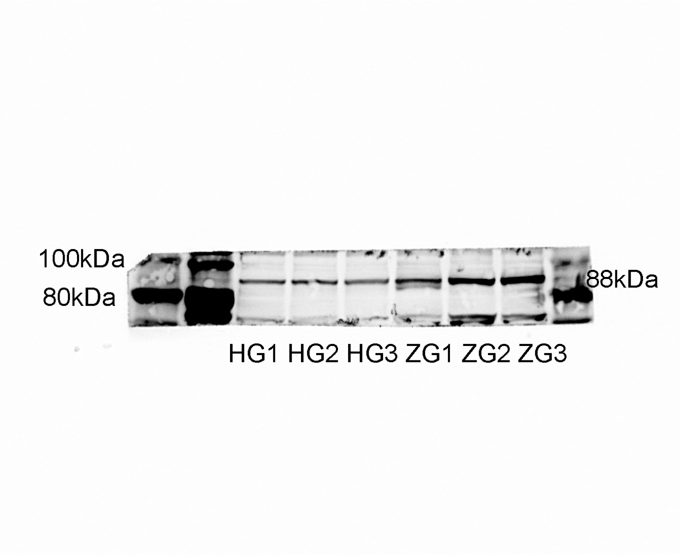

Figure S2. Original Western blot figure of gene CPT1A.

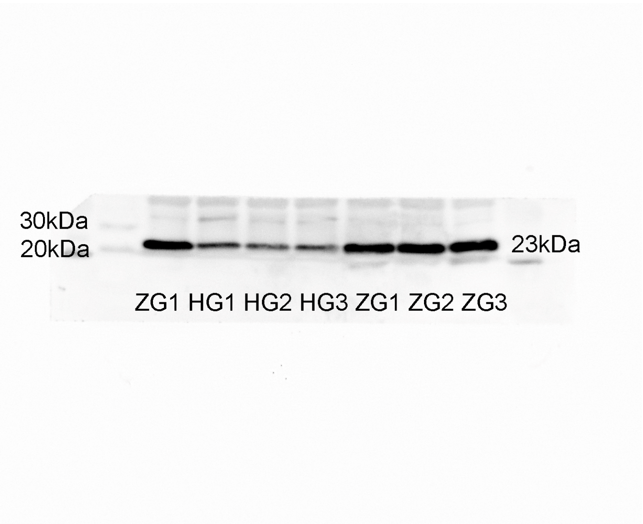

Figure S3. Original Western blot figure of gene FGF21.

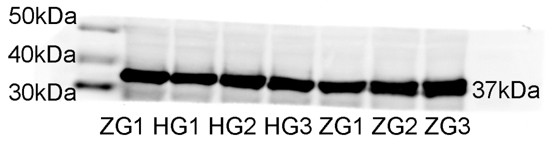

Figure S4. Original Western blot figure of gene GAPDH.
